# Evaluation of the Importance of Stopping Elderly Accidents, Deaths, and Injuries (STEADI)–Based Factors in Wearable Fall Risk Assessment: Secondary Data Analysis

**DOI:** 10.2196/93877

**Published:** 2026-06-09

**Authors:** Nozhan Ghoreishi, Stella Ansah, Jiayu Lu, Wei Lu, Sanghee Moon, Ferdinand Delgado, Diliang Chen

**Affiliations:** 1 Department of Electrical and Computer Engineering University of New Hampshire Durham, NH United States; 2 Department of Computer Science Keene State College Keene, NH United States; 3 Department of Kinesiology University of New Hampshire Durham, NH United States

**Keywords:** fall risk assessment, fear of falling, older adults, smart insole, STEADI, Stopping Elderly Accidents, Deaths, and Injuries, wearable sensors

## Abstract

**Background:**

Falls among older adults are a growing and costly public health problem that often leads to mobility decline and loss of independence. Although clinical frameworks such as the Centers for Disease Control and Prevention’s (CDC) Stopping Elderly Accidents, Deaths, and Injuries (STEADI) initiative recommend multifactor screening (gait, balance, strength, fear of falling, and fall history), most wearable fall risk assessment systems rely on a small set of risk factors (typically gait), which creates a gap between clinical practice and automated wearable assessment.

**Objective:**

This study aims to evaluate the importance of STEADI-based fall risk factors and provide design guidance for clinically compatible wearable fall risk assessment systems.

**Methods:**

We created a dataset of 24 older adults (10 low fall risk and 14 high fall risk) from a publicly available plantar pressure dataset of 48 participants by retaining only those with consistent fall risk labels based on both the Berg Balance Scale and the Timed Up and Go test. A total of 18 features were extracted to quantify gait, strength, balance, fear of falling, and fall history. Random forest (RF) models were trained with leave-one-subject-out cross-validation to assess fall risk. Importance of STEADI-based factors was assessed by two methods: (1) estimating Shapley Additive Explanations values based on a single RF model trained on all features; and (2) training 5 separate RF models, each on 1 STEADI factor category, and comparing their fall risk classification accuracies.

**Results:**

In this secondary analysis, the RF model trained on all features achieved a subject-level accuracy of 87.53% (95% CI 75%-100%). Shapley Additive Explanations analysis identified the right foot flat phase ratio (fear of falling feature) as the highest-ranked feature, followed by maximum right forefoot ground reaction force (strength feature), whereas traditional gait features did not appear in the top 10. The 5 separate RF models trained on individual STEADI-based factor categories showed a similar trend in mean participant-level accuracy: fear of falling, 87.59% (95% CI 75%-100%); strength, 79.18% (95% CI 62.5%-95.83%); balance, 70.5% (95% CI 50%-87.5%); gait 70.81% (95% CI 54.17%-87.5%); and fall history 62.37% (95% CI 50%-75.1%). However, paired comparisons did not show statistically significant differences in accuracy between the gait model and the models trained on other factors.

**Conclusions:**

These preliminary results show that commonly overlooked nongait factors are potentially as informative as gait, although clear superiority was not demonstrated in this dataset. The novel foot flat phase ratio ranked higher than all other evaluated features, which showed the value of domain knowledge–informed feature engineering. These preliminary findings indicate that nongait STEADI factors merit consideration in the design of wearable fall risk assessment systems.

## Introduction

Falls are the leading cause of fatal and nonfatal injuries among older adults in the United States [1]. In 2022, falls accounted for approximately 48% of all injury-related deaths among adults aged 65 years or older [1,2]. Each year, falls lead to nearly 3 million emergency department visits and 1 million hospitalizations among older adults [2]. Many older adults who experience falls develop a fear of falling, which can reduce their independence, restrict their mobility, and limit their social interaction [3]. The annual cost of treating nonfatal fall-related injuries among older adults has increased from US $50 billion in 2015 to US $80 billion in 2020 [4] and is projected to exceed US $101 billion by 2030 [5].

To avoid the serious consequences of falls, proactive detection and mitigation of fall risks have been recognized as effective measures and recommended by the Centers for Disease Control and Prevention (CDC). Different from fall prediction or fall detection, fall risk assessment identifies an individual’s risk of falling and helps guide targeted interventions to reduce future falls. The Stopping Elderly Accidents, Deaths, and Injuries (STEADI) initiative, developed by the CDC, is one of the most widely used tools for fall risk assessment. It begins with 3 key screening questions related to history of falls, fear of falling, and unsteadiness, followed by further assessment of gait, strength, and balance. Although the STEADI enables a comprehensive fall risk assessment, its practical application is limited because it is time consuming [6-11] and is typically conducted in clinical settings that may not fully reflect everyday behavior [12-14].

**Figure 1:**
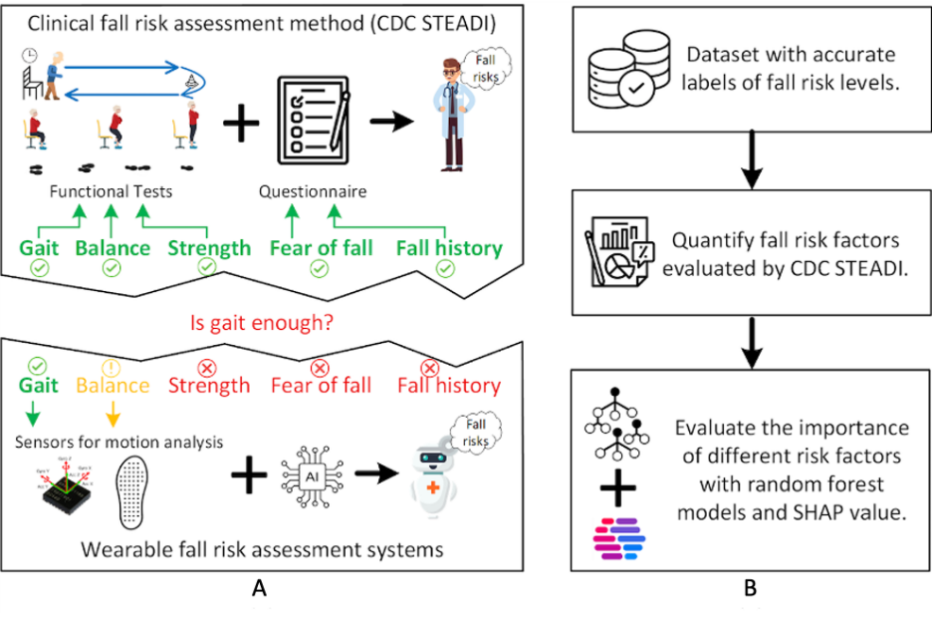
(A) The research question, “Is gait enough?” is motivated by the mismatch between fall risk factors evaluated in clinical assessment methods (Stopping Elderly Accidents, Deaths, and Injuries [STEADI]) and wearable fall risk assessment systems; (B) overview of the proposed method for evaluating the importance of STEADI-based fall risk factors for wearable fall risk assessment. CDC: Centers for Disease Control and Prevention; SHAP: Shapley Additive Explanations.

To address the limitations of clinical assessments, new technologies, especially wearable systems, have been proposed to enable automatic fall risk assessment in free-living environments. However, most existing wearable systems mainly rely on gait-related features, which creates a disconnect from clinical practice. As shown in Figure 1A, clinical methods such as STEADI rely on a comprehensive evaluation of gait, balance, strength, fear of falling, and fall history to assess fall risk. However, existing wearable assessment systems rely on an incomplete set of risk factors (typically gait) extracted from sensor data. For example, inertial measurement unit (IMU)–based wearable technologies have been widely explored for fall risk assessment using measured gait parameters. However, their limitations in measuring strength-related factors prevent them from achieving a comprehensive fall risk assessment required by clinical standards [15,16].

This mismatch between wearable systems and clinical tools raises a research question: “Is gait enough?” To answer this question, this study conducted a comprehensive evaluation of the importance of STEADI-based factors for wearable fall risk assessment. As shown in Figure 1B, this study built a dataset with accurate fall risk labels, quantified fall risk factors evaluated by STEADI, and evaluated the importance of different risk factors using random forest (RF models and Shapley Additive Explanations (SHAP) values.

The contributions of this paper are as follows: first, a new dataset with clinically aligned fall risk labels was built to enable reliable evaluation of the importance of STEADI-based fall risk factors. The Timed Up and Go (TUG) test and the Berg Balance Scale (BBS) are 2 frequently used clinical tests for fall risk assessment. Although neither test alone provides a comprehensive evaluation, their combination covers most of the assessments used by STEADI. This study proposed a new labeling standard that combines TUG and BBS scores to identify participants with high and low fall risk from a public dataset. Second, a comprehensive set of risk factors evaluated by STEADI was quantified. In addition to commonly used features to quantify gait, balance, strength, and history of falls, a novel feature, the foot flat phase ratio (the ratio of the foot flat phase duration to the stance phase duration), was extracted to quantify fear of falling. This feature is informed by cautious gait patterns associated with fear of falling and was found to be the highest-ranked feature in this study. Third, the importance of STEADI-based fall risk factors was comprehensively evaluated to guide the development of wearable fall risk assessment systems. An explainable framework was developed with RF models and SHAP values to quantify the contribution of each factor to fall risk assessment.

## Methods

### Ethical Considerations

The PlantPre dataset used in this study is deidentified and has been released under the Apache-2.0 license. Ethical approval for the collection of the PlantPre dataset was obtained by Hu et al [17] from the Medical Aircraft Ethics Committee of the First Affiliated Hospital of Jinan University (approval number: KY-2020-099).

### Preparing the Dataset With Accurate Fall Risk Labels

**Figure 2:**
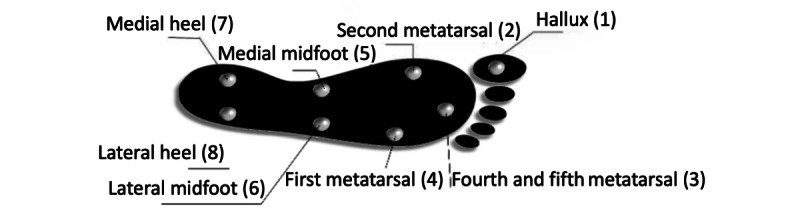
Location of pressure sensors in the smart insole system.

To reliably evaluate the importance of different factors for fall risk assessment, a dataset with accurate labels of participants with high and low fall risk is essential. In this study, a new dataset was prepared based on a publicly available dataset (PlantPre) that includes plantar pressure, fall history, TUG test score, BBS score, body weight (BW), and other variables collected from 48 older adults [17]. The plantar pressure data were collected with smart insoles during 2-3 minutes of normal level-ground walking. As shown in Figure 2, each smart insole has 8 sensors for measuring pressure under the foot.

The TUG test and BBS are widely used for fall risk assessment [18,19]. During the TUG test, the patient is observed and timed while rising from an armchair, walking 3 m, turning, walking back, and sitting down again. A TUG score exceeding a threshold (eg, 13.5 seconds) is used to identify individuals at higher risk of falling [20]. The BBS is a widely used clinical test comprising 14 tasks to assess both dynamic and static balance [21]. A BBS score below a specific threshold (eg, 36) usually indicates high fall risk [22]. However, neither the TUG test nor the BBS alone is sufficient to reliably identify older adults with high risks of falls [23,24].

**Figure 3:**
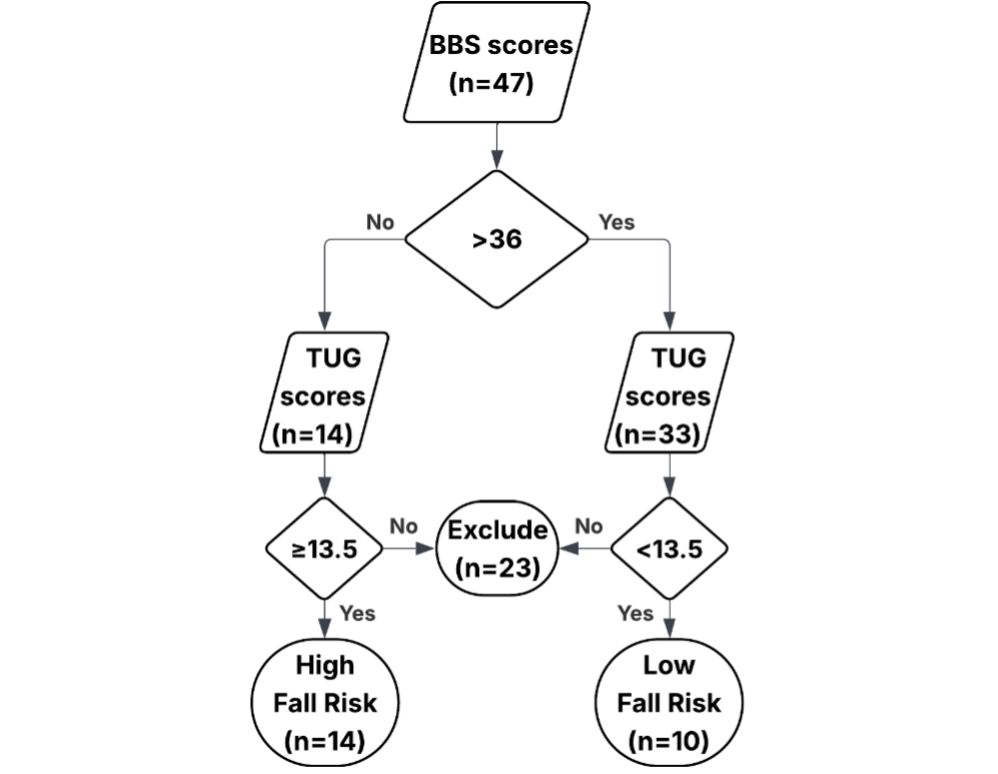
The process used to label participants with high and low fall risk. BBS: Berg Balance Scale; TUG: Timed Up and Go.

To enhance the accuracy of identifying participants with high or low fall risk, reduce noisy fall risk labels, and provide a more reliable basis for the evaluating the importance of STEADI-based factors, a new labeling method that integrates both the BBS and TUG scores was introduced. Firstly, participants were independently labeled based on the BBS and TUG score. Participants with a BBS score ≤36 were classified as high risk, whereas those with a BBS score >36 were classified as low risk [22]. Similarly, participants with a TUG score ≥13.5 seconds were labeled as high fall risk, whereas those with TUG score <13.5 seconds were labeled as low fall risk [20]. Previous research has shown the effectiveness of these thresholds for reliable fall risk assessment [20,22]. Participants whose labels agreed across both tests were then retained, whereas those with conflicting labels were excluded. This approach addressed the limitations of labeling based on a single test and provided a more reliable fall risk labeling. The flowchart of the proposed participant labeling method is illustrated in Figure 3.

Because 1 participant did not have a recorded history of falls, which was 1 of the fall risk factors evaluated in this study, that participant was excluded from this study. Therefore, the total number of participants shown in Figure 3 was reduced to 47. As shown in Figure 3, a total of 23 participants were excluded, and the final dataset prepared for this study comprised 24 participants, with 10 labeled as low fall risk and 14 as high fall risk. As shown in Table 1, the TUG score, BBS score, and fall history of each selected participant were listed. The final dataset of 24 participants had no missing values for any variable used in this study.

**Table 1:** Participant information in the prepared dataset.

Fall risk level and participant ID		TUG^a^ score	BBS^b^ score	Fall history
**Low fall risk**				
	1	8.78	54	No
	2	9.22	54	No
	3	9.41	56	No
	4	10.01	54	No
	5	10.37	54	No
	6	10.5	52	No
	7	11.12	54	No
	8	12.19	50	No
	9	12	37	No
	10	12.93	47	No
**High fall risk**				
	11	21.85	29	Yes
	12	24.68	34	Yes
	13	20.94	28	No
	14	22.03	30	No
	15	22.04	31	No
	16	31.72	35	No
	17	22.35	28	No
	18	29.54	32	Yes
	19	50.37	36	No
	20	25.52	25	No
	21	33.3	28	Yes
	22	45.18	30	No
	23	35.56	27	Yes
	24	33.4	20	No

^a^TUG: Timed Up and Go.

^b^BBS: Berg Balance Scale.

### Quantifying STEADI-Based Fall Risk Factors

#### Overview

According to the STEADI initiative, gait, balance, strength, fall history, and fear of falling are critical factors for fall risk assessment. In this study, a comprehensive set of features was extracted from the prepared dataset to quantify gait, balance, strength, fall history, and fear of falling. The strength and fear of falling features used in this study are pressure-derived proxies rather than direct clinical measures such as dynamometry-measured strength. Table 2 summarizes all the extracted features and their definitions. The total ground reaction force (GRF) was computed as the sum of the pressure measured by all sensors. Subregional (ie, hindfoot, midfoot, and forefoot) GRFs were obtained by summing the pressure readings from sensors within each specific foot region. To account for differences in BW, each participant’s GRF was normalized to BW. The normalized total GRF was then segmented into individual gait cycles using an adaptive threshold method [25]. In total, 3556 gait cycles were detected across 24 participants and were used to extract features to quantify fall risk factors.

**Table 2:** Extracted features for quantifying Stopping Elderly Accidents, Deaths, and Injuries (STEADI)–based fall risk factors.

Category and gait parameters		Description
**Gait features**		
	Gait cycle time (s)	Time duration between 2 consecutive occurrences of any gait event (eg, heel strike) for the same foot. This parameter was calculated for both feet.
	Step time (s)	Time duration from the heel strike of one foot to that of the other foot. This parameter was calculated for both feet.
	Stance time ratio (%)	Length of the interval from heel strike to toe off of the foot in a stride divided by the gait cycle time of the same stride. This parameter was calculated for both feet.
	Swing time ratio (%)	Length of the interval from toe off in a stride to heel strike of the next stride of the foot divided by the gait cycle time of the same stride. This parameter was calculated for both feet.
	Single support time ratio (%)	Length of the interval during which only one foot is in contact with the ground divided by the gait cycle time. This parameter was calculated for both feet.
	Double support time ratio (%)	Length of the interval during which both feet are in contact with the ground divided by the gait cycle time. This parameter was calculated for both feet.
**Strength-related features**		
	Median weight acceptance rate (BW/s^a^)	Median rate of change of the GRF^b^ from heel strike to the weight acceptance peak of the GRF. This parameter was calculated for both feet.
	Median push-off rate (BW/s)	Median rate of change of the GRF from the push-off peak to toe off. This parameter was calculated for both feet.
	Maximum hindfoot GRF (BW^c^)	Maximum force measured by all sensors in the hindfoot region. This parameter was calculated for both feet.
	Maximum midfoot GRF (in BW)	Maximum force measured by all sensors in the midfoot region. This parameter was calculated for both feet.
	Maximum forefoot GRF (in BW)	Maximum force measured by all sensors in the forefoot region. This parameter was calculated for both feet.
**Balance-related features**		
	Absolute CoP^d^ range (cm)	Absolute difference between the maximum and minimum CoP during a gait cycle. This parameter was calculated for the ML^e^ direction and the AP^f^ direction of both feet.
	Average CoP rate of change (cm/s)	Average CoP change during a gait cycle. This parameter was calculated for the ML direction and the AP direction of both feet.
	Maximum CoP rate of change (in cm/s)	Maximum CoP changes during a gait cycle. This parameter is calculated for the ML direction and the AP direction of both feet.
**Fear-of-falling–related features**		
	Foot flat phase ratio (%)	Length of the interval when both hindfoot and forefoot are in contact with the ground, divided by the length of the interval from the heel strike to toe off of the foot during a gait cycle. This parameter was calculated for both feet.
	Heel strike to foot flat phase start (s)	Length of the interval between heel strike and foot flat start within a gait cycle. This parameter was calculated for both feet.
	Foot flat phase end to toe off (s)	Length of the interval between foot flat end and toe off within a gait cycle. This parameter was calculated for both feet.
**History-of-falls–related features**		
	Fall History	A binary variable indicating whether the individual experienced a fall within 1 year before data collection. A value of 1 denotes a history of falling, and a value of 0 denotes no fall history.

^a^BW/s: body weight/second.

^b^GRF: ground reaction force.

^c^BW: body weight.

^d^CoP: center of pressure.

^e^ML: mediolateral.

^f^AP: anteroposterior.

#### Gait Features

**Figure 4:**
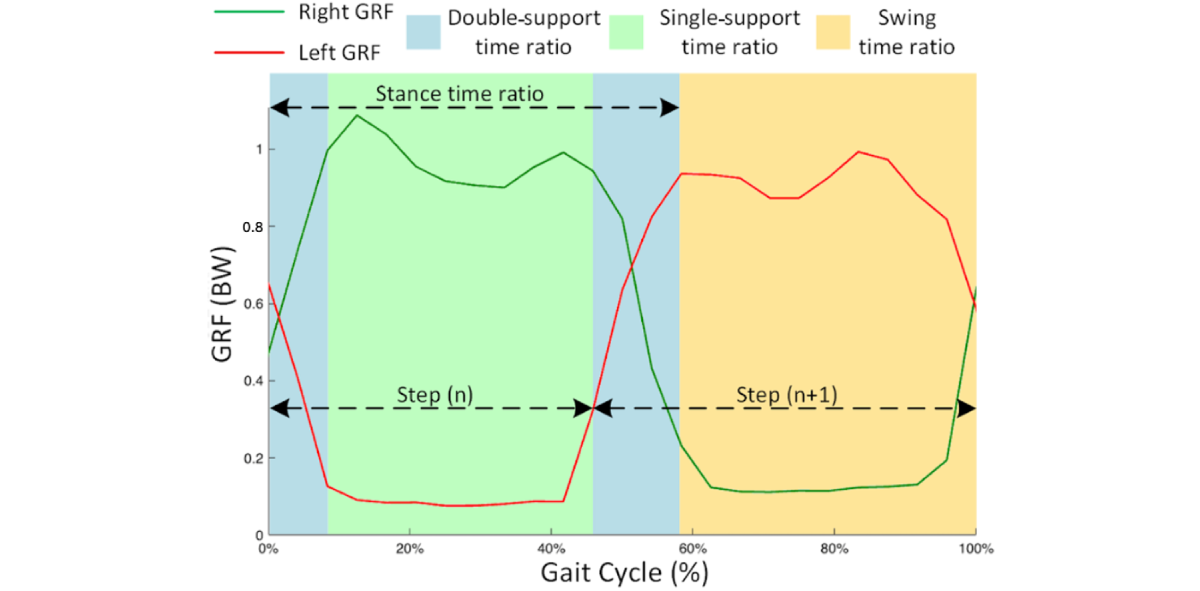
Gait features extracted for a right foot gait cycle. BW: body weight; GRF: ground reaction force.

Gait is recognized as a major fall risk factor among older adults [26]. To quantify gait-related fall risk factors, a total of 6 features (gait cycle time, step time, stance time ratio, swing time ratio, single support time ratio, and double support time ratio) were extracted from each gait cycle. Swing and stance phases were identified using a dynamic threshold proposed in previous research [25]. Figure 4 and Table 2 show the gait features extracted from each gait cycle.

#### Strength-Related Features

**Figure 5:**
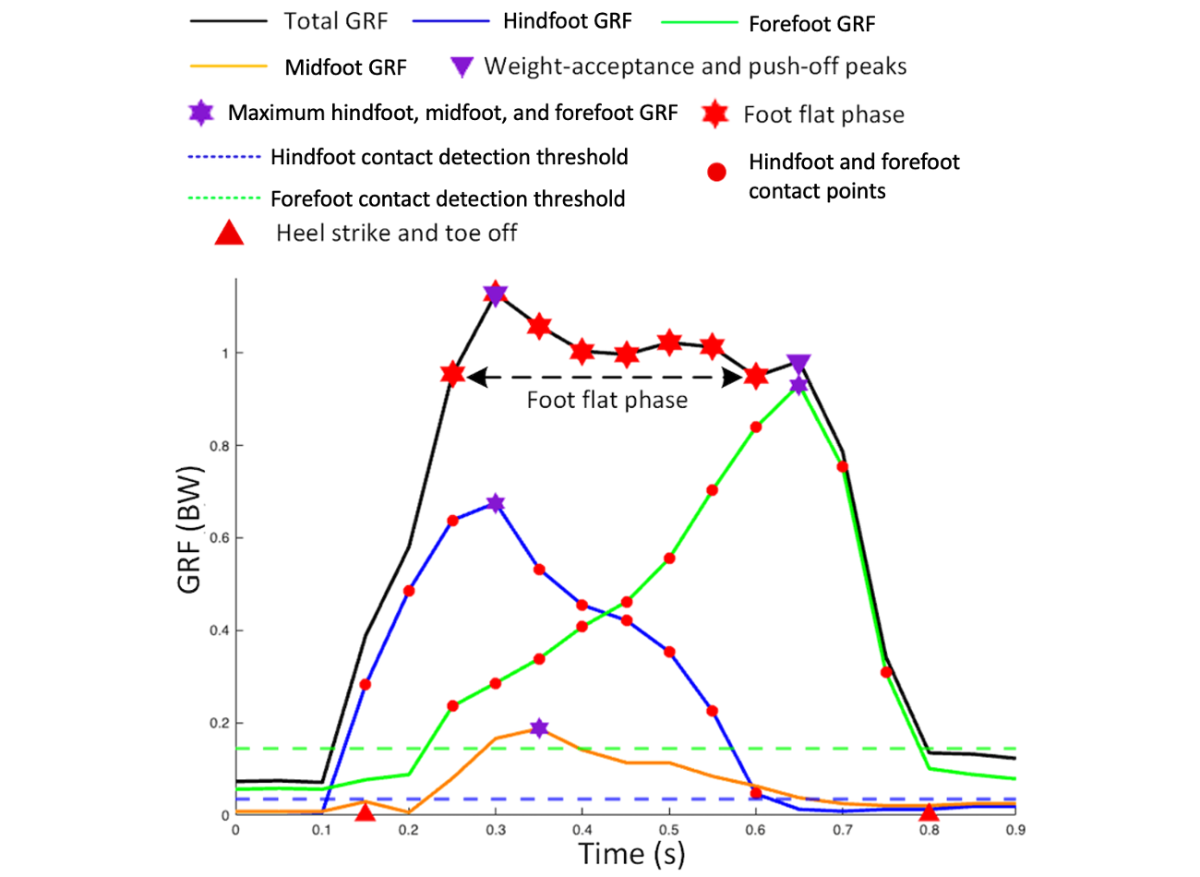
Illustration of key parameters for extraction of strength-related and fear-of-falling–related features. BW: body weight; GRF: ground reaction force.

Strength-related features extracted from plantar pressure during walking are strong indicators of fall risk [27]. In this study, as shown in Figure 5 and Table 2, a total of 5 strength-related features were extracted from plantar pressure [28,29]. In addition to the maximum GRF in the hindfoot, midfoot, and forefoot (purple stars in Figure 5), the median rate of change of the GRF was calculated over 2 intervals: from heel strike to the weight acceptance peak (median weight acceptance rate), and from the push-off peak to toe off (median push-off rate). Zero crossings of the GRF rate-of-change signal were used to identify these peaks. The peaks closest to heel strike and toe off were designated as the weight acceptance and push-off peaks (purple triangles in Figure 5), respectively. In gait cycles with only 1 detected peak, the peak was used to calculate both parameters.

#### Fear-of-Falling–Related Features

Older adults who experience fear of falling often adopt a cautious gait pattern with significant reductions in various parameters [30,31]. While most studies used conventional gait parameters such as stance time and double-support time to quantify fear of falling, these features have not shown significant contributions to fall risk assessment [31]. To more accurately quantify the cautious gait pattern, the stance phase was divided into 3 subphases: heel strike, foot flat, and toe off. People with cautious gait tend to adopt a flat-foot landing strategy that is characterized by a longer foot flat phase duration but shorter intervals from heel strike to foot flat and from foot flat to toe off [32]. Therefore, 3 features were extracted: the ratio of foot flat phase duration to stance phase duration, the time interval from heel strike to the start of the foot flat phase, and the time interval from the end of the foot flat phase to toe off.

The foot flat phase within each gait cycle is defined as the interval during which both hindfoot and forefoot subregions are in contact with the ground [33]. Inspired by previous studies, dynamic GRF thresholds (blue and green dashed lines in Figure 5) were used to detect ground contact of both hindfoot and forefoot regions [25]. As shown in Figure 5, samples in the foot flat phase are marked with red stars, and the times of heel strike and toe off are marked with red triangles on the left and right, respectively.

#### Balance-Related Features

In this study, features extracted from the center of pressure (CoP) were used to quantify balance because of their high reliability and accuracy [34]. The CoP is defined as the location at which pressure is concentrated and is calculated by multiplying each sensor’s coordinates by its respective pressure reading and normalizing by the total GRF. The CoP in the anteroposterior (AP) and mediolateral (ML) directions was calculated in equation 1.



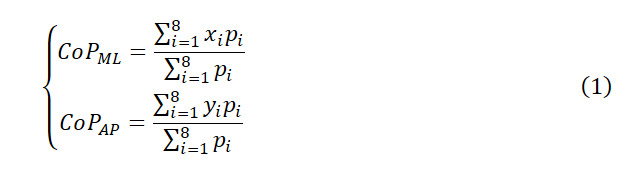



In this equation, CoP_AP_ and CoP_ML_ indicate the CoP in the AP and ML directions, respectively. x_i_ and y_i_ and p_i_ are the coordinates and the pressure readings of sensor i, respectively. CoP variations and rate of change have been shown to be effective for assessing balance [35,36]. In this study, absolute variations in CoP and the mean and maximum rate of change of CoP were extracted for each gait cycle in the ML and AP directions. CoP variations in the ML direction provide information about lateral sway during each gait cycle, whereas AP CoP changes provide information about weight-transfer patterns throughout each gait cycle.

#### History of Fall Feature

History of fall is a binary variable indicating whether the individual experienced 1 or more falls in the past 12 months. A value of 0 indicates no fall history, whereas a value of 1 indicates at least 1 fall during this period.

### Evaluating Importance of Different Fall Risk Factors

To evaluate the importance of different fall risk factors, two methods were implemented (1) training a single RF model using all quantified fall risk factors and evaluating the importance of individual risk factor using SHAP values; and (2) training 5 separate RF models, each using 1 of the 5 types of risk factors, with achieved fall risk classification accuracy used to determine the relative importance of each type of risk factors [37]. Each RF model consisted of 100 decision trees and used the Gini impurity criterion for node splitting. The minimum number of samples required to split an internal node and to form a leaf node was set to 2 and 1, respectively. Following standard RF practice, trees were grown to full depth without a maximum depth constraint. No feature selection was performed. Because class imbalance in the dataset was modest, no explicit class weighting or resampling was applied during training.

To avoid participant-specific overfitting and ensure the achieved accuracy generalizes to unseen individuals, we implemented a leave-one-subject-out cross-validation (LOSO-CV), in which the model was trained on 23 participants and tested on the remaining 1 participant. Traditional validation approaches that shuffle and split samples across all participants can place data from the same participant in both training and testing sets. This can cause the model to rely on memorized participant-specific feature patterns rather than fall-related feature patterns, leading to overestimated testing accuracy. By excluding the testing participant’s data from the training process, LOSO-CV evaluates the true performance of the model in fall risk assessment. Within each LOSO-CV fold, the training and testing data were standardized using the mean and SD computed from the training set only, which prevented information leakage.

To quantify individual feature contributions, SHAP values were used [38]. This method uses a game-theoretic approach to estimate each feature’s impact on the model’s output. SHAP values were first computed for each gait cycle of the held-out test participant of each LOSO-CV fold. For each feature, absolute SHAP values were then averaged across all gait cycles of the held-out participant in that fold to obtain 1 fold-level feature importance value. Finally, these fold-level values were summarized across the 24 LOSO-CV folds using the mean and SD.

## Results

### Overview

This section presents the performance of the RF model for classifying individuals with low and high fall risks, analyzes feature contributions using SHAP values, and evaluates the importance of different types of STEADI-based factors for fall risk assessment.

### Performance of RF Model With all STEADI-Based Features for Fall Risk Assessment

Table 3 summarizes the model’s classification accuracy for each participant in the dataset. Using all extracted features as inputs, the RF model achieved an overall accuracy of 84.93% in fall risk classification across all gait cycles. Although 3556 gait cycles were available in the dataset, cycles within a participant are not independent. Therefore, the participant was treated as the primary unit for performance analysis. To determine each older adult’s final risk of falling, majority voting across all their gait-cycle classifications was used. A participant was considered correctly classified if at least 50% of their gait-cycle classifications matched the ground-truth fall risk label. The RF model achieved a participant-level accuracy of 87.5% in identifying older adults’ risk of falling.

To quantify the uncertainty of model performance, we generated 2000 stratified participant-level bootstrap resamples with replacement from the full LOSO-CV predictions to estimate the mean and 95% CI of the model’s accuracy, sensitivity, specificity, receiver operating characteristic area under the curve, and Brier score, as shown in Table 4.

**Table 3:** Gait-cycle number and fall risk classification accuracy of each individual.

Participant ID	Number of gait cycles, n	Gait-cycle-level accuracy (%)	Participant-level accuracy (%)
1	154	69.48	100
2	150	82	100
3	143	93.71	100
4	132	87.88	100
5	130	96.92	100
6	152	90.79	100
7	148	97.3	100
8	133	74.44	100
9	139	91.37	100
10	153	92.81	100
11	141	100	100
12	187	96.79	100
13	139	100	100
14	129	41.86	0
15	149	28.19	0
16	188	55.85	100
17	98	97.96	100
18	207	100	100
19	211	99.05	100
20	154	46.1	0
21	98	100	100
22	89	100	100
23	159	100	100
24	173	100	100
Total	3556	84.93	87.5

**Table 4:** Performance of the random forest model trained with all features for fall risk assessment.

Metrics	Values, mean (95% CI)
Accuracy, %	87.53 (75-100)
Sensitivity, %	79.10 (57.14-100)
Specificity, %	100 (100-100)
ROC-AUC	0.99 (0.96-1.00)
Brier score	0.07 (0.02-0.12)

### Importance of Different Types of Fall Risk Factors Based on SHAP Values

**Figure 6:**
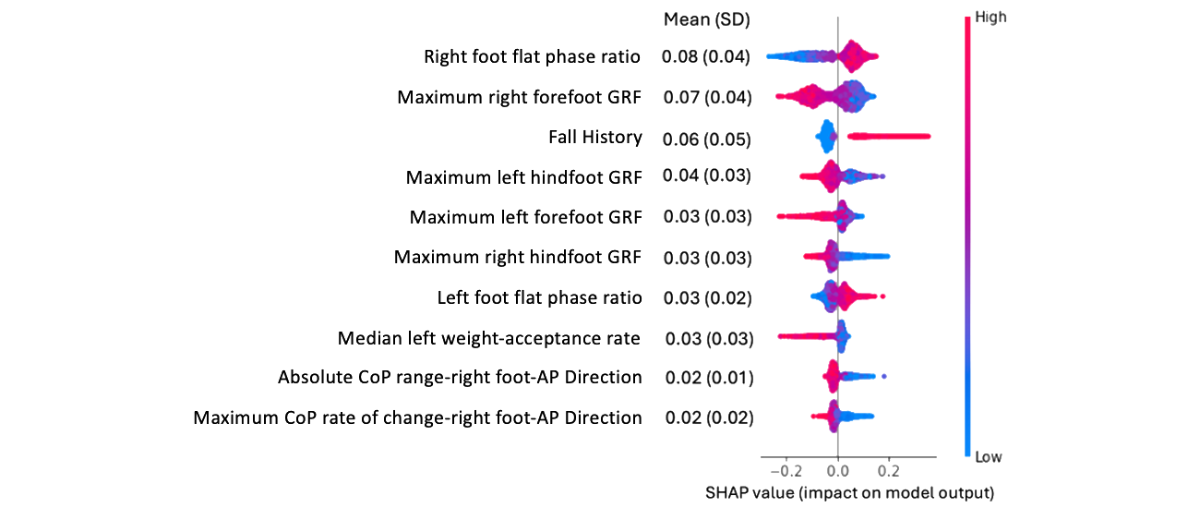
The top 10 most important features for fall risk assessment based on Shapley Additive Explanations (SHAP) values. AP: anteroposterior; CoP: center of pressure; GRF: ground reaction force.

Figure 6 summarizes the top 10 features in predicting the risk of falls based on SHAP values. Mean and SD of absolute SHAP values are presented in Figure 6. Among all STEADI-based features, the “right foot flat phase ratio” has the highest rank. According to Figure 6, a higher foot flat phase ratio leads to the prediction of high fall risk, which aligns with clinical findings that longer foot flat phases characterize cautious gait patterns associated with fear of falling and high fall risk [32]. The “maximum right forefoot GRF” ranked second, with higher values predicting lower fall risk. This is consistent with clinical knowledge, as a higher maximum forefoot GRF indicates greater lower-limb strength, which contributes to better gait stability and balance and consequently a lower risk of falls. Fall history ranked third, with more previous falls predicting higher fall risk. This is consistent with clinical practice, where fall history is recognized as an indicator of risk of falls. However, because the dataset includes only 24 participants, the relative ordering should be interpreted as preliminary.

### Importance of Different Types of Fall Risk Factors Based on Classification Accuracy

To further illustrate the importance of different types of STEADI-based factors for fall risk assessment, 5 separate RF models were trained, each using features from only 1 type of factor listed in Table 2. The classification accuracy achieved by each model was used to determine the importance of the corresponding type of fall risk factor.

Table 5 shows the classification accuracy achieved by each category of fall risk factor. Overall, fear-of-falling–related features, strength-related features, balance-related features, gait features, and the history-of-fall feature achieved accuracies of 75.68%, 72.44%, 68.34%, 66.31%, and 62.5%, respectively. This ranking is mostly consistent with the result in the previous section “Importance of Different Types of Fall Risk Factors Based on SHAP Values.” However, because the dataset includes only 24 participants, the relative ordering should be interpreted as preliminary.

To quantify the uncertainty of model performance, we generated 2000 stratified participant-level bootstrap resamples with replacement from the full LOSO-CV predictions to estimate the mean and 95% CI of the model’s accuracy, sensitivity, specificity, receiver operating characteristic area under the curve, and Brier score, as shown in Table 6.

To further evaluate the significance of differences between gait features and other fall risk factor features, a paired bootstrap comparison was performed. The mean and 95% CI of the accuracy differences between the model trained on gait features and the models trained on other fall risk factor features were calculated across 2000 stratified participant-level bootstrap resamples. As shown in Table 7, the mean paired accuracy differences for “fear of falling–gait,” “strength–gait,” and “balance–gait” are all positive. Since the corresponding 95% CIs indicate that these paired differences were not statistically significant, the results indicate that nongait factors are potentially as informative as gait, although clear superiority was not demonstrated in this dataset.

**Figure 7:**
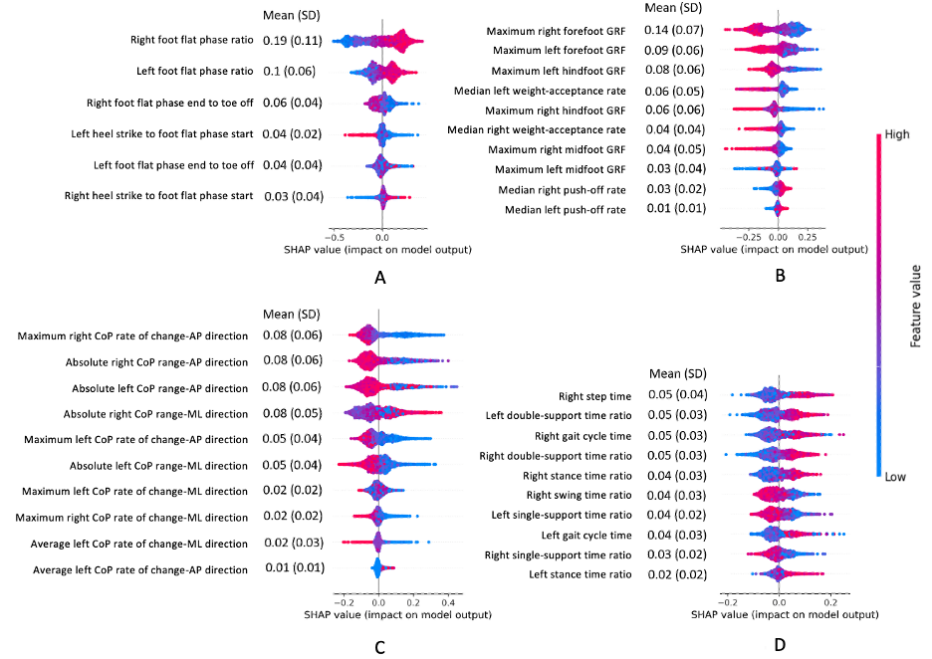
Top features in different categories of Stopping Elderly Accidents, Deaths, and Injuries (STEADI)–based fall risk factors: (A) fear-of-falling–related features, (B) strength-related features, (C) balance-related features, and (D) gait-related features. AP: anteroposterior; CoP: center of pressure; GRF: ground reaction force; ML: mediolateral.

To provide more insight into the importance of individual features within each category of fall risk factors, SHAP values for the features are shown in Figure 7. According to Figure 7A, older adults with a higher risk of falling showed a longer foot flat phase ratio. This aligns well with clinical findings that older adults with a fear of falling adopt a cautious gait with longer period for the foot flat phase [32].

As shown in Figure 7B, maximum GRF of the forefoot and hindfoot were identified as the most important strength features, with higher maximum GRF indicating a lower risk of fall. This is consistent with clinical findings, as maximum forefoot and hindfoot GRF correspond to peak forces during toe off and heel strike, respectively, and higher forces reflect greater lower-limb strength for controlling walking posture and maintaining stability. Similarly, the weight acceptance rate, which correlates with the hindfoot GRF, was also ranked highly in the list. The lower ranking of maximum midfoot GRF is expected, as it reflects forces during the relatively static foot flat phase rather than the critical dynamic heel strike and push-off phases. The low ranking of “median push-off rate” was unexpected. The reason for this may be the hysteresis effect of the pressure sensor during the push-off phase, which potentially leads to an underestimation of this feature.

Additionally, among the balance-related features (Figure 7C), the maximum CoP velocity in the AP direction was identified as the most important feature for fall risk assessment. As indicated by the SHAP values, older adults at higher risk of falling exhibited lower maximum COP velocities in the AP direction, which reflects a reduced walking speed. This is consistent with clinical findings that older adults at high risk of fall are associated with slower walking speeds.

Finally, as shown in Figure 7D, step time and double-support time were the most important gait features for fall risk assessment. Based on the SHAP analysis, a higher double-support time and step time indicate a higher risk of falling. This result is consistent with clinical studies, where older adults at a higher risk of falling have prolonged double-support and step time [31].

**Table 5:** Accuracy of different types of factors for fall risk assessment.

Participant ID	Fear-of-falling–features (%)	Strength-related features (%)	Balance-related features (%)	Gait features (%)	History-of-fall features (%)
1	44.81	83.77	38.31	11.04	100
2	60	58	87.33	74.67	100
3	87.41	41.26	82.52	56.64	100
4	65.91	84.85	49.24	49.24	100
5	71.54	96.92	16.15	53.85	100
6	76.32	91.45	63.16	38.16	100
7	96.62	67.57	86.49	88.51	100
8	50.38	78.95	69.92	54.89	100
9	49.64	47.48	35.97	66.91	100
10	74.51	52.94	62.75	43.14	100
11	98.58	63.83	75.89	92.2	100
12	50.8	18.72	56.15	78.61	100
13	77.7	100	97.84	86.33	0
14	86.05	81.4	34.11	21.71	0
15	74.51	8.72	32.89	74.5	0
16	74.47	63.83	91.49	47.34	0
17	98.98	96.94	98.98	93.88	0
18	87.92	94.2	28.99	89.37	100
19	69.19	96.68	100	37.44	0
20	94.81	42.86	85.06	98.7	0
21	46.94	90.82	75.51	89.8	100
22	87.64	100	94.38	100	0
23	100	100	100	91.19	100
24	92.49	100	83.24	79.19	0
Overall	75.68	72.44	68.34	66.31	62.5

**Table 6:** Performance of the random forest model trained with individual feature set for fall risk assessment, reported as mean (95% CI).

Feature set	Accuracy, % (95% CI)	Sensitivity, %, (95% CI)	Specificity %, (95% CI)	ROC-AUC (95% CI)	Brier score (95% CI)
Fear of fall features	87.59 (75-100)	93.02 (78.57-100)	80 (50-100)	0.97 (0.9-1.0)	0.09 (0.05-0.13)
Strength features	79.18 (62.5-95.83)	78.53 (57.14-100)	80.09 (50-100)	0.88 (0.71-1.00)	0.14 (0.06-0.23)
Balance features	70.5 (50-87.5)	78.19 (57.14-100)	59.73 (30-90)	0.82 (0.63-0.96)	0.16 (0.09-0.25)
Gait features	70.81 (54.17-87.5)	78.15 (57.14-100)	60.53 (30-90)	0.83 (0.64-0.98)	0.17 (0.10-0.25)
History-of-fall features	62.37 (50-75.10)	35.5 (14.29-57.5)	100 (100-100)	0.68 (0.57-0.82)	0.37 (0.21-0.50)

**Table 7:** Paired accuracy differences between gait and other fall risk factors, reported as mean (95% CI).

Factors	Values, mean (95% CI)
Fear of falling–gait	0.17 (−0.04 to 0.42)
Strength–gait	0.08 (−0.17 to 0.38)
Balance–gait	0.00035 (−0.21 to 0.21)
Fall history–gait	−0.08 (−0.25 to 0.12)

## Discussion

### Overview

This paper presented a comprehensive study to quantify the importance of STEADI-based factors for wearable fall risk assessment. Based on a newly developed dataset with accurate fall risk labels and a comprehensive feature set that quantifies STEADI-based fall risk factors, the importance of different STEADI-based factors was evaluated using RF models and SHAP analysis. This section discussed the new knowledge generated from this study, provides design guidance for the development of wearable fall risk assessment systems, and specifies the limitations of this study and plans for future work.

### STEADI-Based Factors Other Than Gait Are Also Important for Wearable Fall Risk Assessment

Although clinical studies have shown that gait, balance, strength, fear of falling, and history of falling are important for fall risk assessment [39-41], most wearable systems still mainly rely on gait features for fall risk assessment [42-44]. The importance of nongait fall risk factors in wearable fall risk assessment has not been comprehensively evaluated.

Choi et al [43] developed a model to predict the TUG score using gait features extracted from 3D acceleration data collected from pelvis and both feet during normal walking, and then used the predicted TUG scores for fall risk assessment. Maiora et al [42] used IMU sensors to collect motion data during the TUG test and built convolutional neural networks and recurrent neural network models based on gait features extracted from the IMU data for fall risk assessment. Agrawal et al [44] proposed a smart insole–based system for fall risk assessment. They used a commercial smart insole with 5 force-sensitive resistance sensors to collect dynamic plantar pressures from 5 foot locations: big toe, medial forefoot, lateral forefoot, midfoot, and heel. The fall risk labels were generated using the TUG score with a threshold of 13.5, and the RF model achieved an accuracy of 81% in fall risk classification. Although the smart insole has the potential to quantify a comprehensive set of fall risk factors, this study focused on gait and balance features only without exploring the importance of different types of factors for fall risk assessment.

This study provides preliminary evidence to address this gap by comprehensively evaluating the importance of STEADI-based factors for wearable fall risk assessment. The results show that nongait factors are potentially as informative as gait, although clear superiority was not demonstrated in this dataset. The exclusion of these important factors may lead to unreliable fall risk assessment results. This finding provides preliminary design guidance on the necessary factors that should be considered when developing wearable fall risk assessment systems.

### Effective Features Can Be Generated From Domain Knowledge

To build the connection between clinical practice and wearable fall risk assessment systems, it is important that the outputs of wearable systems are explainable using clinical knowledge. One solution is to ensure that predicted fall risks are described using clinically meaningful features. This requires developing features based on domain knowledge.

This study showed that effective features can be generated from domain knowledge. For example, based on the domain knowledge that older adults with fear of falling usually adopt a cautious gait with a flat-foot walking pattern, new features specifically focused on the foot flat phase were extracted. The foot flat phase ratio feature was shown to be the most effective feature for fall risk assessment, as shown in Figure 6. This new feature was more effective than many widely used fear-of-falling features, such as stance time and double-support time, which were not ranked in the top 10 features in Figure 6. Compared with stance time and double-support time, the foot flat phase ratio captures how the stance phase is redistributed around foot contact and push-off, rather than simply prolonged duration. This makes it more sensitive to changes in landing strategy and weight-transfer behavior, which may reflect a cautious walking pattern associated with fear of falling. This finding contributes to the knowledge base on effective features to quantify fear of falling and shows the value of domain knowledge in developing new features for fall risk assessment.

### Limitations and Future Work

Although this study quantified different fall risk factors and presented a preliminary evaluation of their importance in fall risk assessment, its scope is limited by the existing dataset, which will be addressed by future studies.

First, although the analysis included 3556 gait cycles, these gait cycles were collected from only 24 participants. Therefore, the effective independent sample size is determined by the number of participants rather than the number of gait cycles. The limited number of participants may affect the stability of feature-importance rankings when generalizing to a broader population. Therefore, the main contribution of this preliminary study is not the feature-importance ranking but the finding that STEADI-based factors beyond gait features may also contribute meaningfully to fall risk assessment. Given the limited participant-level sample size and wide CIs, the findings should be interpreted as preliminary. Additionally, although the class imbalance in the dataset was modest (14 high-risk vs 10 low-risk participants), it may still bias the model toward the majority class and influence feature-importance estimates. In future work, we will build a new more balanced dataset with more participants to further explore the relative importance of STEADI-based factors.

Second, the gait, strength, balance, and fear-of-falling–related features were extracted only from the plantar pressure data collected using a smart insole with 8 pressure sensors. One potential shortage is that 8 sensors may not provide enough spatial resolution to quantify different risk factors with optimal accuracy. Additionally, although temporal gait parameters were extracted from plantar pressure data, spatial gait parameters such as stride length and gait speed could not be extracted for analysis. This may lead to an underestimation of the contribution of gait-related features. In the future, we plan to address these limitations by using the smart insole developed in our laboratory. This insole has 96 pressure sensors uniformly distributed across the insole, and an accurate IMU sensor integrated into it. It has been comprehensively validated and successfully applied in various research projects [25,33,45-52]. With this smart insole, we will build a new dataset by collecting comprehensive data from more older adults, enabling a more accurate and comprehensive evaluation of the importance of different fall risk factors.

Third, to reliably evaluate the importance of different factors for fall risk assessment, the dataset used in this study was built by excluding participants whose TUG and BBS labels disagreed. Although this method reduces label noise, the resulting dataset may not fully reflect the heterogeneity of real clinical populations and may reduce ecological validity. Therefore, the reported model performance may overestimate the accuracy achievable in a heterogeneous clinical population. Additionally, the estimated importance of STEADI-based factors may not fully generalize to participants whose BBS and TUG assessments disagree. Validation in a larger and more heterogeneous cohort, including discordant cases, is an important direction for future work.

Participant-level classification was obtained by majority voting across all gait cycles of each participant. A participant was considered correctly classified if at least 50% of their gait-cycle classifications matched the ground-truth fall risk label. However, the stability of this approach depends on the number of gait cycles available for each participant and may be reduced when the number of cycles is relatively small or when the proportion of positive classifications is close to 50%. Therefore, participant-level classifications near the decision boundary should be interpreted with caution.

### Conclusion

This study provides a preliminary evaluation of the importance of STEADI-based factors for wearable fall risk assessment. The study quantified a comprehensive set of STEADI-based factors and introduced a novel domain-knowledge–informed feature, the foot flat phase ratio. The key finding is that commonly overlooked nongait factors are potentially as informative as gait, although clear superiority was not demonstrated in this dataset. These preliminary findings indicate that nongait STEADI factors merit consideration in the design of wearable fall risk assessment systems.

## Abbreviations

AP: anteroposterior
BBS: Berg Balance Scale
BW: body weight
CDC: Centers for Disease Control and Prevention
CoP: center of pressure
GRF: ground reaction force
IMU: inertial measurement unit
LOSO-CV: leave-one-subject-out cross-validation
ML: mediolateral
RF: random forest
SHAP: Shapley Additive Explanations
STEADI: Stopping Elderly Accidents, Deaths, and Injuries
TUG: Timed Up and Go
